# Natural Killer Cell Receptors and Cytotoxic Activity in Phosphomannomutase 2 Deficiency (PMM2-CDG)

**DOI:** 10.1371/journal.pone.0158863

**Published:** 2016-07-14

**Authors:** Roberto García-López, María Eugenia de la Morena-Barrio, Laia Alsina, Belén Pérez-Dueñas, Jaak Jaeken, Mercedes Serrano, Mercedes Casado, Trinidad Hernández-Caselles

**Affiliations:** 1 Departamento de Bioquímica, Biología Molecular B e Inmunología, Facultad de Medicina, IMIB-University of Murcia, Murcia, Spain; 2 Centro Regional de Hemodonación, Servicio de Hematología y Oncología Médica, Hospital Universitario Morales Meseguer, IMIB-Arrixaca, Murcia, Spain; 3 CIBERER, Valencia, Spain; 4 Sección de Alergia e Inmunología Clínica, Hospital Sant Joan de Déu, Barcelona, Spain; 5 Departamento de Neurología Infantil, Hospital Sant Joan de Déu, Barcelona, Spain; 6 Center for Metabolic Diseases, Universitair Ziekenhuis Gasthuisberg, KULeuven, Leuven, Belgium; 7 Departamento de Bioquímica Clínica y Neuropediatría, Hospital Sant Joan de Deu CIBERER-ISCIII, Barcelona, Spain; Institut Pasteur, FRANCE

## Abstract

**Background:**

PMM2-CDG is the most common N-glycosylation defect and shows an increased risk of recurrent and/or severe, sometimes fatal, infections in early life. We hypothesized that natural killer (NK) cells, as important mediators of the immune response against microbial pathogens and regulators of adaptive immunity, might be affected in this genetic disorder.

**Objective:**

To evaluate possible defects on PMM2-CDG NK peripheral blood cell number, killing activity and expression of membrane receptors.

**Methods:**

We studied fresh and activated NK cells from twelve PMM2-CDG cells. The number and expression of lymphoid surface receptors were studied by flow cytometry. The NK responsiveness (frequency of degranulated NK cells) and killing activity against K562 target cells was determined in the NK cytotoxicity assay.

**Results:**

We found an increase of blood NK cells in three patients with a severe phenotype. Two of them, who had suffered from moderate/severe viral infections during their first year of life, also had reduced T lymphocyte numbers. Patient activated NK cells showed increased expression of CD54 adhesion molecule and NKG2D and NKp46 activating receptors. NKp46 and 2B4 expression was inversely correlated with the expression of NKG2D in activated PMM2-CDG cells. Maximal NK activity against K562 target cells was similar in control and PMM2-CDG cells. Interestingly, the NK cell responsiveness was higher in patient cells. NKG2D and specially CD54 increased surface expression significantly correlated with the increased NK cell cytolytic activity according to the modulation of the killer activity by expression of triggering receptors and adhesion molecules.

**Conclusions:**

Our results indicate that hypoglycosylation in PMM2-CDG altered NK cell reactivity against target cells and the expression of CD54 and NKG2D, NKp46 and 2B4 activating receptors during NK cell activation. This suggests a defective control of NK cell killing activity and the overall anti-viral immune response in PMM2-CDG patients. The present work improves our understanding of the immunological functions in PMM2-CDG and possibly in other CDG-I types.

## Introduction

Congenital disorders of glycosylation (CDG) are rare genetic diseases caused by defective glycosylation of glycoproteins and glycolipids. Some 100 CDG have been reported. These disorders show an extremely broad clinical spectrum that can affect nearly all organs and systems, including immunity, with degrees of severity that range from early death to very mildly affected adults [1, 2].

PMM2-CDG, one of the most prevalent CDG, is an autosomal recessive defect of phosphomannomutase 2 due to mutations in *PMM2* [3]. Both cell surface and secreted glycoproteins are affected. PMM2-CDG patients show numerous neurological features (such as psychomotor disability, axial hypotonia, retinitis pigmentosa, ataxia, stroke-like episodes, epilepsy and peripheral neuropathy), as well as other organ involvement (gastro-intestinal dysfunction, skeletal abnormalities, hypogonadism, immunodeficiency a. o.). The phenotype expression ranges from near-normal to very severe, with an increased mortality in the first years due to vital organ involvement or severe infection [1, 2].

Immunological function in PMM2-CDG has been partially studied. Blank et al. [4] analysed adhesion molecules in two patients and found that patient neutrophils had normal rolling on artificial endothelium but diminished chemotaxis while expressing comparable levels of adhesion molecules (such as Mac-1, L-selectin, P-selectin glycoprotein ligand-1 (PSGL-1) and platelet endothelial cell adhesion molecule-1 (PECAM-1)). Their most significant finding was a poor humoral response after vaccination against several microorganisms. Bergmann et al. [5] found a diminished α2,6 and an increased α2,3 sialylation in EBV transformed lymphoblastic B cells from PMM2-CDG patients maybe affecting interactions of CD22, an important regulator of B cell activation, with its ligands. More recently, the adhesion molecule CD54 (ICAM-1) has been found diminished in various subtypes of CDG-I patient fibroblasts and in N-glycosylation-deficient Chinese hamster ovary (CHO) cells [6]. We have detected abnormal glycosylation of CD16 and CD14 GPI-anchored proteins in PMM2-CDG patient neutrophils and monocytes. This potentially affects the affinity of immune receptors to their ligands and conditions the immune response [7]. Up to now, there is no further investigation concerning the function of other leukocytes such as NK cells or NK cell expressing molecules in CDG patients.

Natural killer (NK) cells are important mediators of the immune response against microbial pathogens and tumor cells. They can also control immune responses by mediating the killing of autologous or allogeneic normal cells. NK cells can regulate specific humoral and cell-mediated immunity by producing a great amount of cytokines (IFN-γ, TNF-α, GM-CSF, IL-10) and chemokines (IL-18, CCL2, MCP-1). NK cell immunoregulatory and cytotoxic functions are controlled by a repertoire of activating and inhibitory receptors. Among activating receptors NK cells use receptors such as CD16, NKG2D, 2B4, CD226, NKp80, NKp46, NKp44 and NKp30, most of them implicated in recognition of viral products and/or self-molecules induced in conditions of cellular stress. The inhibitory signals are mediated mainly by HLA class I-binding receptors, including killer cell Ig-like receptors (KIRs), CD94/NKG2A, and leukocyte Ig-like receptor B1 (LILR-B1) [8, 9]. NK cell responsiveness is assessed during development using both activating and inhibitory receptors to ensure self-tolerance while allowing efficacy against infections and tumors (reviewed by Vivier et al. [10]). Additional molecules such as the adhesion molecules LFA1 and ICAM1 increase conjugation and intracellular signals during the interplay between NK and target cells [11].

In the present work, we studied peripheral blood cytotoxic cell numbers and NK cell phenotype and cytotoxic function mainly in expanded quiescent NK cells from PMM2-CDG patients. A significant positive correlation was found between the elevated expression of CD54 and the elevated reactivity against target cells on patients NK cells. An increase in NKG2D expression correlated with increased cytotoxicity and was accompanied by a skewed expression of the NKp46 and 2B4 activating receptors. In addition, a simultaneous increase of blood NK cell numbers and a decrease of T lymphocyte numbers was observed in two patients suffering from moderate/severe viral infections. Altogether, our results suggest a defective control of NK cell responsiveness in these cells that may impair anti-viral immune responses in PMM2-CDG and thus possibly in other CDG-I patients.

## Material and Methods

### 1. PMM2-CDG patients and controls

Twelve PMM2-CDG patients, aged 1–35 years, were enrolled. Venous blood was collected into citrate-tubes and delivered within 24–48 hours by express courier at room temperature to Murcia, Spain, where all further studies were done. Blood samples were drawn during non-infectious periods.

Thirty healthy controls, including 3 children, were also enrolled in this study. Controls, patients, and relatives were fully informed of the aim of this study, which was performed according to the declaration of Helsinki, as amended in Edinburgh in 2000. This study obtained approval from the University of Murcia Ethics Committee. Written informed consent was obtained from all participants, including parental consent in the case of enrolled children.

### 2. Stimulation and expansion of peripheral blood lymphocytes (PBL)

In order to obtain high amounts of NK cells from the patients, PBL were stimulated and expanded by co-culturing whole blood (4 μl/well) with irradiated allogeneic cells in complete tissue culture medium (TCM: RPMI 1640 plus 10% fetal calf serum and 1% penicillin/streptomycin) supplemented with IL-2 (100 U/ml) as described [12]. After 9–10 days stimulation, cells were transferred to 24 well plates and maintained in TCM supplemented with 100 U/ml of rIL-2 until quiescence (low or null expression of the activation surface antigen CD25) which occurred approximately three-four weeks after stimulation. Then cells were phenotyped and assayed for cytotoxic activity.

### 3. Flow cytometry analysis

Flow cytometry was the method of choice to study the expression of several surface activating or inhibitory receptors on patients stimulated but quiescent cells. For activated T lymphocyte and NK cell evaluation we analysed CD3-FITC, CD16-Cy5, CD54-PE, CD56-PE, Siglec7-PE, CD226-PE (DNAM-1), CD11a, CD50 (ICAM-3), NKG2A (CD159a), 2B4 (CD244), NKG2D (CD314) and NKp46 (CD335) (S1 Table). Stained cells (20,000) were collected using a FACS Scalibur cytometer (Becton-Dickinson, Mountain View, CA, USA). For white blood cells 50,000–100,000 events were collected. Data were further analysed using the CellQuest program (Becton Dickinson) and Flowing Software version 2.5.1, to obtain the mean fluorescence intensity (MFI) and the percentage of different stained populations.

### 4. NK cell function analysis

NK cells cytotoxic activity was assessed by studying both NK cells degranulation (a consequence of cytotoxic activity of killer cells) and target cell killing by co-culturing control or patient quiescent effector (E) cells with the NK cell target K562 (T) at different effector/target (E/T) ratios in a 96 well round bottom plate for 4 hours at 37 °C and 5% CO_2_. Peripheral blood mononuclear cells (PBMCs), purified by discontinuous density gradient in Lymphoprep (Nycomed Pharma, Oslo, Norway), were also proved in the case of patients P2, P5 and the corresponding healthy controls.

To study the frequency of degranulated cells, the number of effector cells was kept constant. After 2 hours of effector and target incubation, anti-human CD107a PE conjugated mAb (eBioscience) and monensin (2 μM final concentration, Sigma-Aldrich) were added to the wells according to [13]. Finally, we stained the cells with anti-CD3 FITC to identify NK and T lymphocytes, fixed the cells (Lyse/Fix buffer, Becton Dickinson) and determined the percentage of degranulated NK cells (CD3^-^CD107a^+^) by flow cytometry.

The frequencies of degranulated NK CD107a^+^ cells (α) at every E/T ratio (R) were calculated and expressed as the percentage of CD107a^+^ NK cells related to the total number of acquired NK cells. All data pairs (αi; Ri) were mean values from measurements performed in two separated wells. Then, the data of α vs R was plotted and a logarithmic curve (y = a ln(x)+b) was fitted to calculate the frequency of degranulated NK cells at a common E/T ratio that was chosen at E/T 1:1. The greatest values for NK degranulation (α_max_) were also obtained as a measurement of the maximal percentage of NK cells that reacts against target cells on each individual cell line. α_max_ was calculated by plotting 1/α vs 1/R (i.e., vs. 1/R (T:E ratio)) and lineal regression analysis, being 1/α_max_ the intersection point of this plot.

We also determined the MFI of CD107a on CD107a^+^ NK cells as a measure of a) the expression of this heavily glycosylated protein and b) the amount of lytic granules discharged by individual degranulating NK cells that can describe their cytolytic activity.

To study target cell killing, K562 target cells were labelled with CFSE (carboxyfluorescein diacetate succinimidyl ester; Invitrogen) and co-cultured (10,000 target cells/well) with variable numbers of effector cells. After 4 hr incubation, cells were washed twice with PBS stained by adding 20 μl/well propidium iodine (PI, Sigma-Aldrich) at 50 μg/ml, then data were acquired by flow cytometry. Percentages of killed targets (CSFE^+^ PI^+^ K562 cells) among CSFE^+^ target cells were plotted against E/T ratio and analysed by linear regression. Percentages of target killing were compared among samples at a common E/T ratio that was chosen at E/T 0.5:1 and was calculated by extrapolation from these plots. In some experiments we measured effector cell viability during the cytotoxicity assay by flow cytometry. Briefly, after 4 hr incubation of effector and target cells, we stained cells with annexin-V-FITC (eBioscience), anti-CD56-PE and anti-CD3 PerCP as described above. Then, data were acquired and percentages of apoptotic NK (CD3- CD56+annexin+) and T (CD3+ annexin+) cells were determined.

To compare NK cells activity from different donors, we normalized to the mean activity of the control samples.

### 5. Statistical analysis

Percentages of MFI values were calculated as reported [7] and are described as mean+/SD. Study groups were compared by Mann-Whitney U test. Pearson test was applied to study the correlation between parameters. P-values <0.05 were considered statistically significant.

## Results

The demographic, genetic and clinical characteristics of these patients are shown in Table 1. S2 Table shows the distribution of the plasma sialotransferrin fractions (with the typical increase of di- and asialotransferrin and decrease of trisialotransferrin, a so-called type 1 pattern). All patients had normal age-matched levels of serum IgG, IgA, IgM, and normal vaccine responses to tetanus, diphtheria and pneumococcus. Patient P1 suffered from severe viral infections (rotavirus (non-vaccine) at 3 months of age, requiring ICU admission for 2 months, complicated with post-natal CMV infection that resolved with gancyclovir treatment). Patient P2 acquired an adenovirus infection al 5.5 months of age, a severe nosocomial rotavirus infection at 12 months of age during an admission for a Gtube placement, requiring ICU admission, and died 2 months later of multiorgan failure. Patient P3 developed a rotavirus enteritis at 5 months of age, with no admission, followed by a RSV bronchiolitis that required ventilatory support and, at 8 months of age, an adenovirus enteritis. Thereafter, he showed recurrent ear infections with rapid response to oral antibiotics. Patients P1 and P3 were vaccinated with MMR without adverse events.

**Table 1:** Clinical, demographic and genetic characteristics of PMM2-CDG patients.

Patient	Nationality	Age (years)	Sex	*PMM2* mutation	Clinical severity	Severe infections (1^st^ year of life).
**P1**	Spain	1	M	E33X, V44A	Severe	**Yes (viral)**
**P2**	Spain	1	F	R141H, V231M	Severe	**Yes (viral)**
**P3**	Spain	4	M	Y64C, R141H	Severe	**Yes (viral)**
**P4**	Spain	4	F	P113L, haploinsufficiency	Severe	No
**P5**	Spain	9	F	P113L, T118S+P184D	Severe	No
**P6**	Czech Republic	5	F	C9Afs, P113L	Moderate	No
**P7**	Belgium	1	M	P113L, T237R	Mild/Moderate	No
**P8**	Belgium	8	M	D188G, V231M	Mild/Moderate	No
**P9**	Belgium	13	M	F119L, R141H	Mild/ Moderate	No
**P10**	Spain	10	F	F157S, R162W	Mild	No
**P11**	Belgium	35	F	P113L, R141H	Mild	No
**P12**	Belgium	35	F	P113L, R141H	Mild	No
**Adult controls**	Belgium, Czech Republic and Spain	40 ±10	19M, 10F	-	-	-
**Pediatric controls**	Belgium and Spain	7 ±1	3M	-	-	-

### T and NK cells frequency in patient peripheral blood

At the time of sample reception, we examined and compared basal frequencies of lymphoid CD3^+^ (T lymphocytes) and CD3^-^CD16^+^ (NK cells) in the blood of PMM2-CDG and control subjects (Table 2**)**. Frequencies of CD3^-^CD16^+^ and CD3^+^ cells in PMM2-CDG patients were similar to control subjects with the exception of the severe patient P2 which showed a high NK/T ratio as a consequence of a notable increase of CD3^-^CD16^+^CD56^+^ NK cells (895 cells/μl) and significant decrease of the amount of CD3^+^ cells (1003 cells/μl). NK/T ratio was also slightly increased in the severe CDG patient P1 due to an increase of CD3^-^CD16^+^ NK lymphoid cells (1368 cells/μl vs 3578 cells/μl CD3^+^ cells). NK cell frequencies were not dependent on age as shown before [14]. Moreover, P2 NK cells subpopulations, analysed according to CD56 and CD16 markers, showed normal levels of CD56 and CD16 antigen expression (not shown) and normal ratios of CD56^dim^ and CD56^brigth^ cells (97.2% and 2.8% respectively among CD3^-^CD56^+^ lymphoid cells).

**Table 2:** Basal numbers of peripheral blood T and NK cells at the time of sample reception.

Patients	CD16^+^ (%)	CD3^+^ (%)	CD16^+^/CD3^+^ ratio
**P1**^a^	21.3	50.8*	0.42*
**P2**^a^	34.3*	37.3*	0.92*
**P3**^a^^,^ ^b^	10.0	58.4^*^	0.17
**P4**^a^	14.3	75.0	0.19
**P5**^a^	3.6*	72.5	0.05
**P6**	6.0	43.5*	0.14
**P7**	6.2	62.9	0.10
**P8**	18.1	76.3	0.24
**P10**	22.9	78.0	0.29
**P11**	16.3	73.8	0.22
**P12**	12.7	74.6	0.17
**Controls**	16.1 ± 7.9	75.8 ± 5.6	0.21 ± 0.1

* Out of range values

^a^ Severe patients

^b^ Data at the age of 4.

### Phenotype of CDG activated NK cells

**Fig 1:**
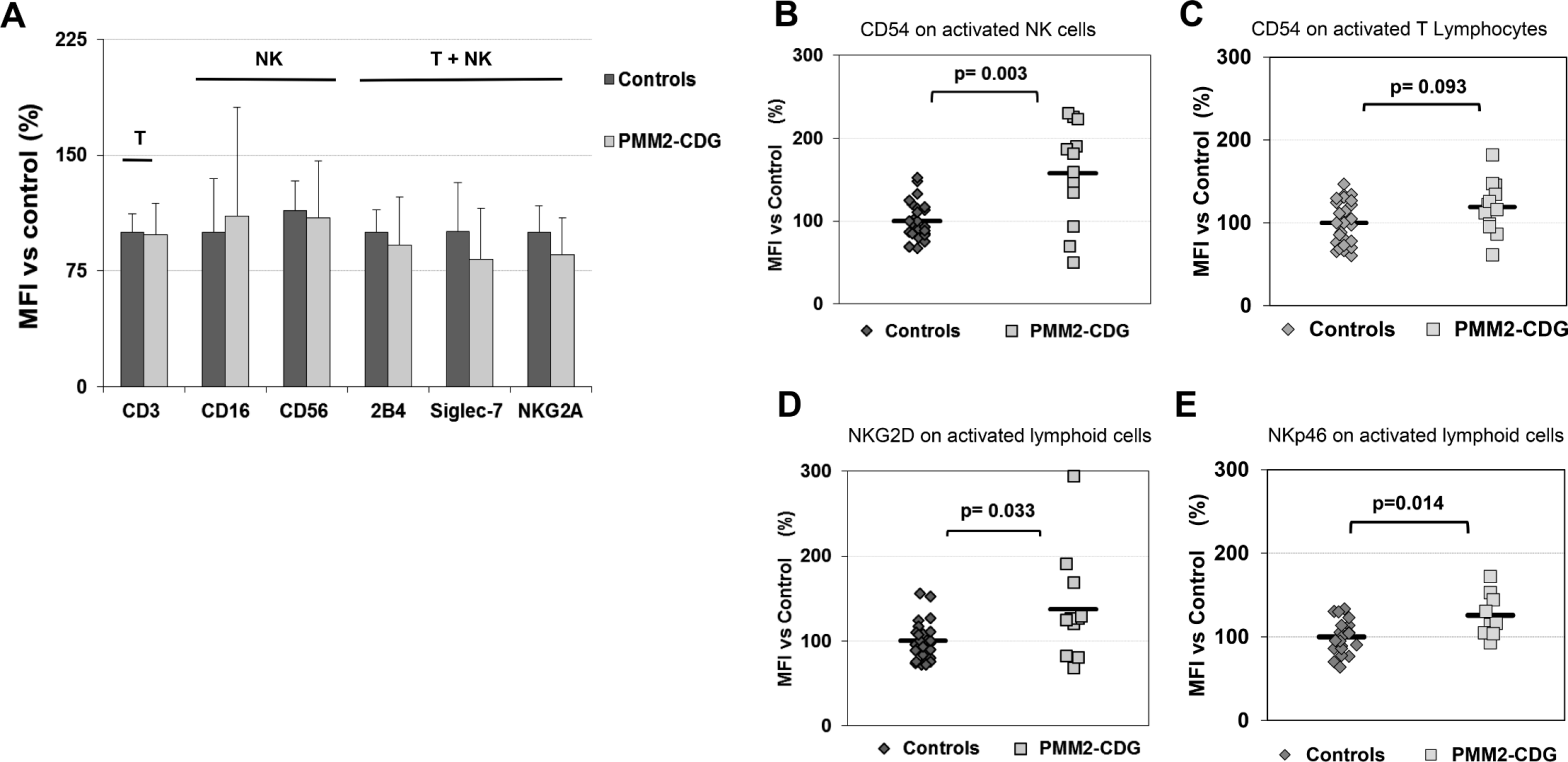
**(A-E) Expression of surface receptors on activated NK and T cells from PMM2-CDG patients and control subjects. (B, C) CD54 (ICAM-1), (D) NKG2D and (E) NKp46 expression.** The study was done by flow cytometry. Values are expressed as % MFI *vs* that observed in control subjects.

Expanded cells after stimulation (T and NK lymphocytes) were studied for the expression of several membrane receptors, including NK inhibitory and activating receptors by flow cytometry and MFI relative percentage determination in order to detect phenotype differences among patients and healthy subjects. In accordance with our previous work [7], CD3 and CD16 surface expression in PMM2-CDG patients was similar to that in control cells. Other receptors such as CD56 or Siglec-7 (inhibitory receptor) in NK cells or 2B4 (activating receptor) or NKG2A (inhibitory receptor) in both T and NK cells, were also similarly expressed in PMM2-CDG and control cells (Fig 1A).

Additionally, we observed an overall significant increase of CD54 adhesion molecule (p = 0.003; n = 12), and NKG2D (p = 0.033, n = 11) and NKp46 (p = 0.014, n = 9) activating receptors expression on the surface of NK cells compared to control individuals (Fig 1B, 1D and 1E). Increased CD54 expression indicated an increased level of activation of PMM2-NK cells [11, 15].

**Fig 2:**
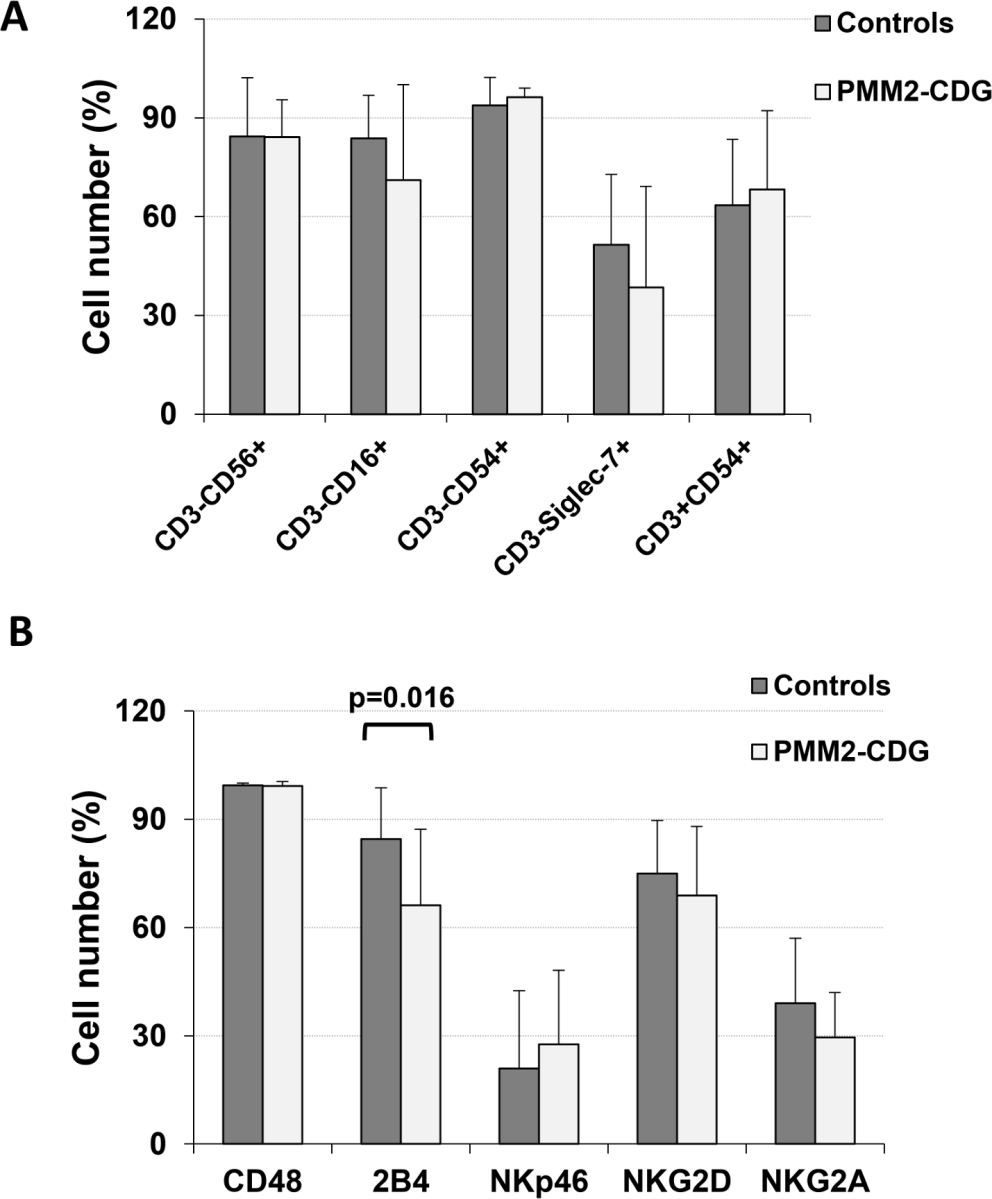
**(A) Percentages of expanded NK (CD3^-^) and T (CD3^+^) cells, indicated as percentages of CD16^+^, CD56^+^, CD54^+^ and siglec-7^+^ subpopulations. (B) Percentages of other expanded lymphoid subpopulations.** The study was done by flow cytometry. Values are expressed as % cells ± SD. The amount of 2B4+ cells was significantly lower (p = 0.016) in expanded activated patient lymphocytes.

The percentages of activated NK cells (CD3^-^) in our cultures, with subpopulations CD16^+^, CD56^+^, CD54^+^ and siglec-7^+^ cells, and of CD54^+^ T cells were not significantly different control cultures (27.6 ± 18.1% and 34.3 ± 25.2% respectively) (Fig 2A). However, the 2B4^+^ subpopulation was significantly decreased (p = 0.016; n = 10) in PMM2-CDG patient expanded cells (Fig 2B).

### CD54, NKG2D and NKp46 expression on lymphoid cells from PMM2-CDG patients

**Fig 3:**
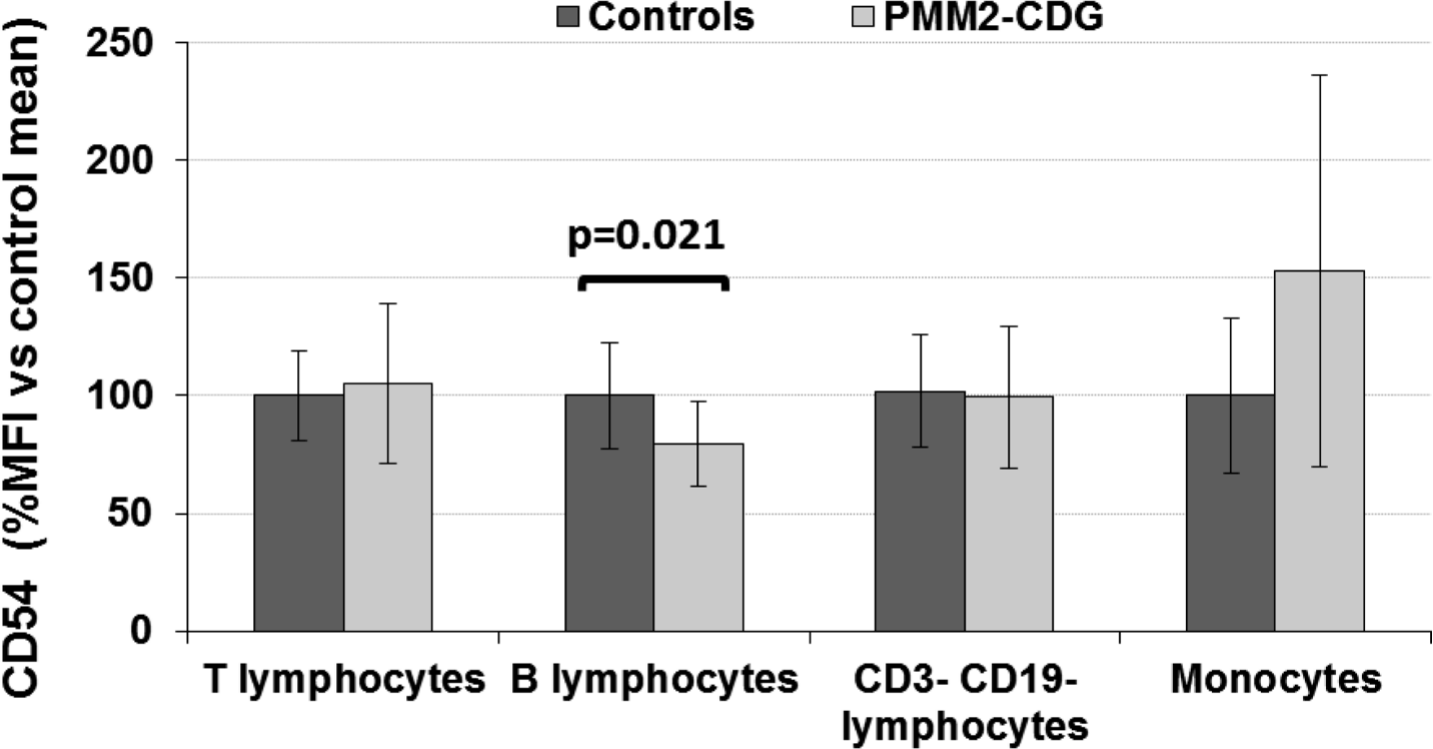
Level of CD54 expression on different blood lymphocyte subpopulations and monocytes in PMM2-CDG patients and control subjects. The study was done by flow cytometry. Values are expressed as % MFI *vs* that observed in controls. CD54 expression was found significantly lower (p = 0.021) in patient B lymphocytes.

Recently, a decreased expression of CD54 has been reported in fibroblasts from several CDG patients [6] and in MPI-CDG mice [16]. To study the basal expression of this marker in our patients we determined CD54 expression on the surface of several blood resting cell populations, i. e., T (CD3^+^), B (CD19^+^), CD3^-^CD19^-^ (probably NK) lymphocytes and monocytes, by flow cytometry. As shown in Fig 3, only B lymphocytes showed significantly lower levels of CD54 constitutive expression in our cohort of patient compared to controls (p = 0.021; n = 10). As we have shown before [7], CD54 levels on neutrophils were similar in PMM2-CDG patients and controls using the same antibody.

Basal expression of NKG2D was found lower in patients P2, P6 and P10 (71.3%, 89.1% and 71.9% respectively *vs* control cells) than in control blood lymphoid cells indicating that NKG2D expression increased upon NK cell activation. Basal expression of NKp46 was very low and of similar intensity in patient and control blood lymphoid cell (not shown).

### Cytotoxic activity in PMM2-CDG activated NK cells

**Fig 4:**
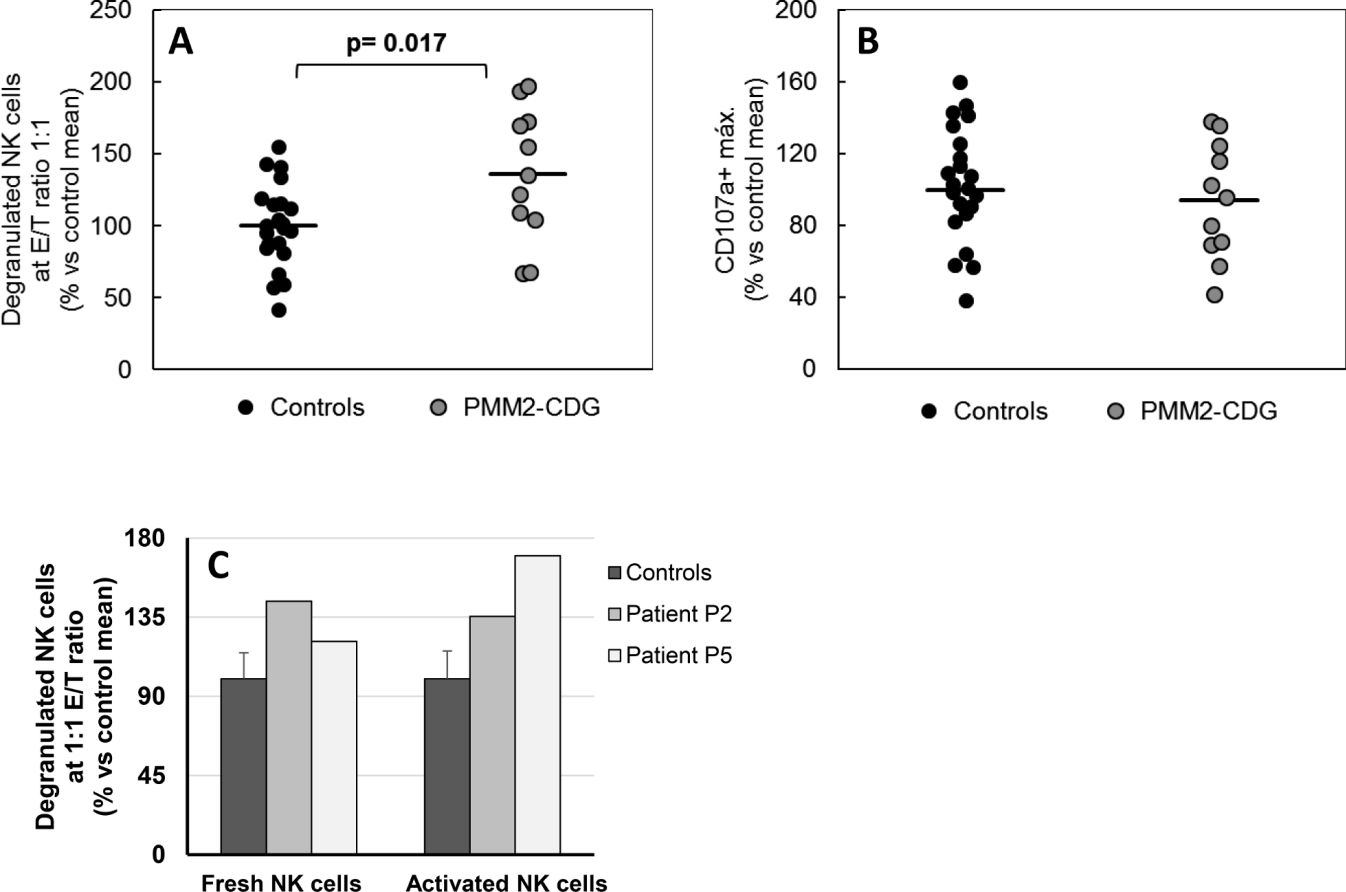
Reactivity of NK cell against K562 target cells. (A) Percentage of degranulated NK cells at 1:1 E/T ratio (see Materials and Methods section). (B) Máximal reactivity of NK cells calculated as the maximal number of degranulated CD107a^+^ NK cells (α_max_). Values from all tested patients and control subjects are shown. Horizontal lines represent the mean value in each study group. (C) Percentage of degranulated NK cells at 1:1 E/T ratio of fresh and activated NK cells from patient P2 (light grey bars), P5 (white bars) and control subjects (dark bars). Standard deviation in control samples is shown.

The overall NK cytotoxic activity against K562 target cells was first evaluated by determining a) the frequency of degranulated NK cells at E/T ratio 1:1 (Fig 4A) and b) the maximal frequency of degranulated CD107a^+^ NK cells (α_max_) (Fig 4B), as indexes that can describe NK cell activation and reactivity [17]. We found that the maximal frequency of degranulated NK cells (α_max,_ i. e. frequency at high E/T ratios) from patients was similar to that of control cells indicating a normal maximal frequency of activated NK cells against K562 targets on patient samples. However, we found that their reactivity, i. e. the frequency of degranulated cells at low E/T ratios such as 1:1 (Fig 4A, and S1 Fig) or lower E/T ratios (not shown), was significantly increased (p = 0.017, n = 11). Reactivity of both fresh and activated quiescent NK cells from patient P2 and P5 was found higher than control NK cells (Fig 4C). Relative reactivity values of P2 and P5 *vs* control NK cells were similar in both fresh and activated quiescent NK cells.

We also studied the fluorescence intensity of externalized CD107a on CD107a^+^ NK cells. CD107a, a highly glycosylated molecule, has a role in the protection of cytotoxic cells from degranulation-associated suicide [18]. CD107a externalization may be used as a measure of the mean lytic granules discharged by individual degranulating NK cells during the cytotoxicity assay [19]. Collectively, we found no significant differences among control and patients cells (S2 Fig) indicating normal granule discharge in patients NK cells. Patient´s (P3, P5 and P6) T and NK cell apoptosis (annexin-V FITC positive cells) was tested during the cytotoxicity assay. Patients showed normal values of apoptotic NK (15.7 ± 3.7% vs 11.7 ± 4.3% in control cells) and T (4.5 ± 3.0% vs 4.6 ± 2.8%) indicating cell protection during cytotoxic activity in patient effector cells.

Finally, the killing activity against the target cell was tested on activated quiescent NK cells from severe patients P3, P5, the mild/moderate patient P9 and their corresponding control cells. Results showed comparable killing activity of patient NK cells and control subjects. Killing activity was coupled with both a normal or, in the case of patient P5, a greater granule discharge or mature active perforin amount [20] (S3 Table).

### Correlations between CD54, NKG2D and NKp46 expression with increased cytolytic activity

**Fig 5:**
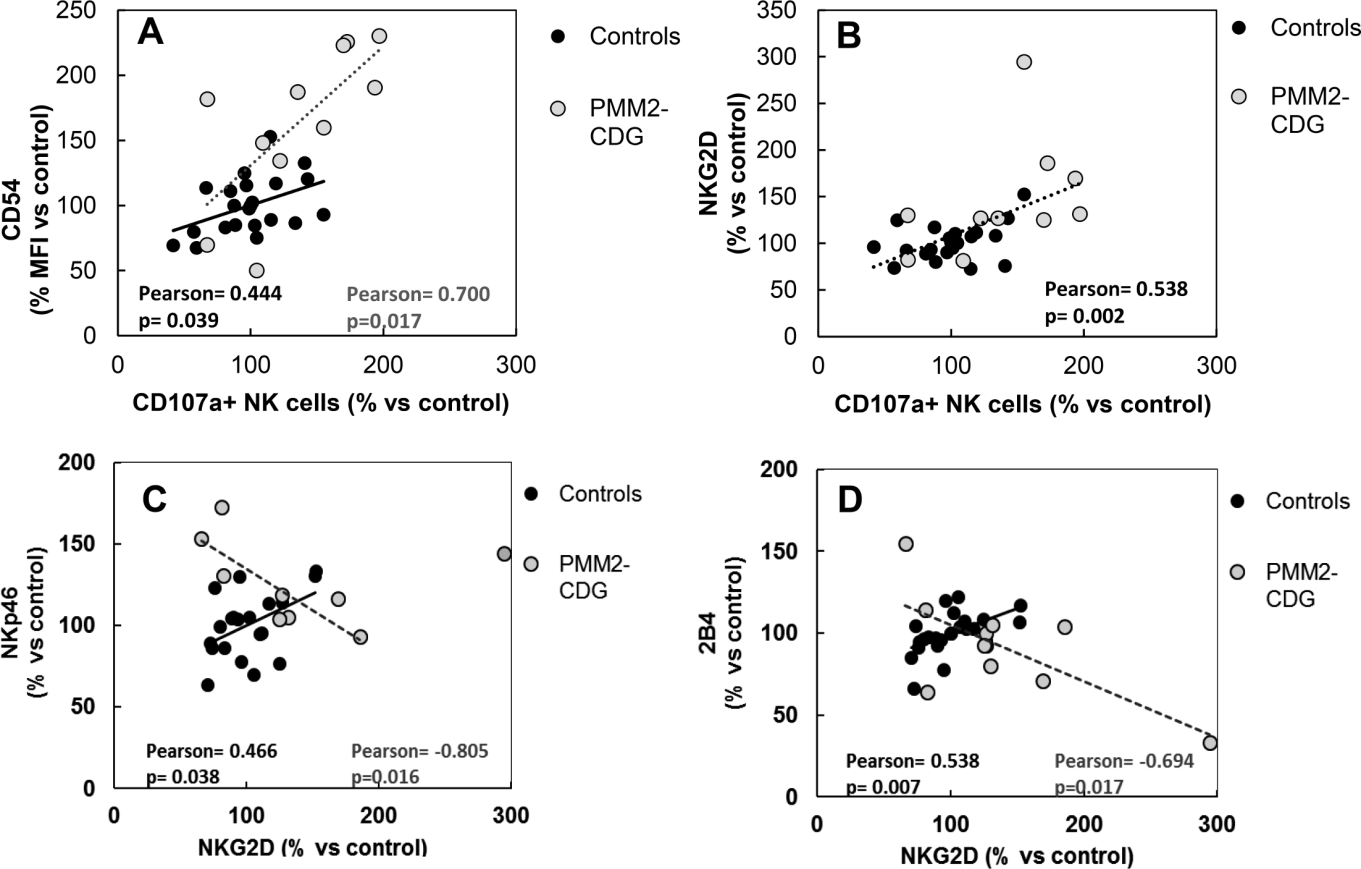
**Correlation of CD54 (A) and NKG2D (B) expression on NK cells according to NK cell reactivity, at E/T 1:1 ratio, in PMM2-CDG patients and healthy subjects. Correlation of NKp46 (C) and 2B4 (D) expression with NKG2D expression.** Values from all patients (light dots and dashed lines) tested and control subjects (dark dots and continues lines) are shown. Lines represent the linear tendency using control and patient values. R^2^ values from linear regression, Pearson correlation indexes and p values are shown in black letters for control values and in grey letters for patient´s values.

The increased frequency of degranulated NK cells could be due to the increased expression of activating receptors implicated on natural cytotoxicity and the increased level of activation of NK cells. Thus, we studied the correlation between NK degranulation frequencies and the levels of expression of different membrane receptors. We found a significant and strong direct correlation between degranulation activity (E/T 1:1) and the expression of CD54 on NK cells from both control (Pearson = 0.444, p = 0.039) and patient cells (Pearson = 0.700, p = 0.017) cells (Fig 5A). PMM2-CDG patients presented the highest values for both frequency of degranulated NK cells and CD54 expression indicating that the expression level of the activation marker CD54 may be related to the increase of degranulation activity in PMM2-CDG patients. Correlation between degranulation frequency and NKG2D expression values from both patients and controls was lower and significant (Pearson = 0.523; p = 0.009). Seven patients out of ten tested presented higher values for NK degranulation and NKG2D expression than control mean values suggesting that the level of expression of NKG2D could also be related to the increased reactivity of the NK cells in PMM2-CDG patients (Fig 5B). Correlations between NK degranulation and NKp46 or 2B4 activating receptor expression were not significant (Pearson = -0.629, p = 0.130 and Pearson = 0.118, p = 0.746 respectively) among patient cells. Moreover, and opposite to controls, we found a strong negative correlation between NKp46 or 2B4 and NKG2D activating receptor expression among patient cells (Fig 5C and 5D) indicating that NKG2D increased expression did not occur along with the increase of NKp46 or 2B4 activating receptors. These results suggest a skewed expression of activating receptors after activation in PMM2-CDG lymphocytes.

Consistent with the findings of previous studies on PMM2-CDG patients, we did not find a correlation of clinical severity or age with any of the studied parameters.

### Hyper-responsive PMM2-CDG NK cells expressed normal levels of CD226 functional marker and CD11a and CD50 adhesion molecules

NK cells functional response is assessed in the bone marrow during differentiation and maturation stages. Hypo- or hyper-responsive NK cells are obtained in the absence of interactions between killer inhibitory or activating receptors and their ligands in both mouse and human (reviewed in [21]). CD226 activating receptor expression has recently been found to correlate with the functional response of normal blood NK cells serving as a marker for the cytolytic potential of human NK cells [22].

In an attempt to understand the observed hyper-responsiveness of patients NK cells we studied the expression of CD226 activating receptor and CD11a/CD18 adhesion molecules, key regulators of NK function with numerous potential glycosylation sites [22]. CD50 (ICAM-3, intercellular adhesion molecule-3) expression was also determined. We found a similar level of expression of CD226 on all fresh and activated NK and T cell subsets in patients P3, P5 and P6 and in control cells. (S4A and S4B Table). Similarly, percentages of patient CD226^+^ blood T (CD3^+^) cells were normal, whereas percentages of CD226^low^ NK (CD3^-^CD19^-^) lymphocytes were higher than control mean in one of two ‘severe’ patients tested. CD11a and CD50 expression was also normal on patient lymphocytes (S5 Table). In conclusion, CD226 expression did not correlate with the higher reactivity displayed by PMM2 NK cells. PMM2 defects may not have a relevant impact on the expression of CD226, CD11a and CD50 NK regulatory molecules.

### NK and TCD8^+^ cell counts showed opposite kinetic profiles in two PMM2-CDG patients with severe phenotype and suffering from viral infections

**Fig 6:**
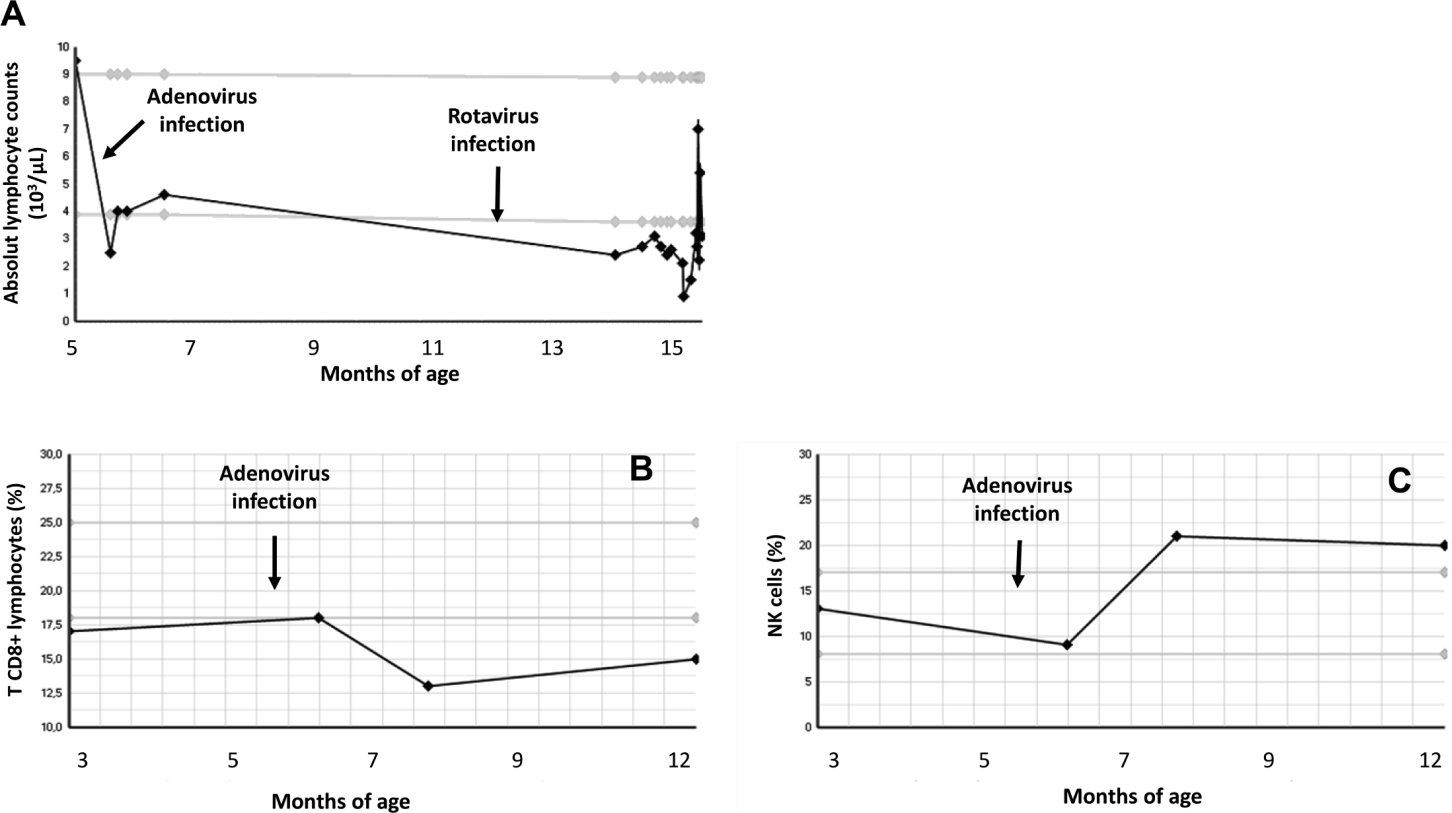
**T lymphocytes (A and B) and NK cell (C) kinetic profile during the first year of life in patient P2.** Black arrows indicate the onset of adenoviral and rotavirus infection in this patient. Light grey horizontal lines represent normal range values.

Recent investigations have shown that NK cells can regulate adaptive immune response against viral infections either by reducing memory T cell numbers, producing T cell suppressor cytokines, etc. [9]. Thus, the elevated basal counts in two severe patients which suffered from severe viral infections during the first year of life and the increased reactivity of NK cells found in PMM2-CDG patients prompted us to study the lymphocyte subset counts over time in patient P2, as shown in Fig 6. In this patient, leukocyte counts decreased after an adenovirus infection at 5.5 months of age and later, after a rotavirus infection at 12 months of age (Fig 6A). Surprisingly, this reduction was associated with a strong decrease of CD8^+^ T cells (Fig 6B) and a notable increase of NK cell counts (Fig 6C). Moreover, we found similar relative levels of TCD8^+^ and NK cells in the blood of patient P3 at the age of 15 months (Table 3). TCD4^+^ and NK cell levels were normal in this patient one year later, in the absence of severe viral infections. At that time, TCD8^+^ cells levels were still low. Altogether, our results suggest that cytotoxic adaptive response may be compromised in these patients at least during their first year of life.

**Table 3:** Absolute and percentage cell counts of peripheral blood lymphocytes in patient P3.

	Absolute counts (cells/μL)			Cell percentages		
	15 months^a^	28 months^a^	Normal values	15 months^a^	28 months^a^	Normal values
**Total lymphocytes**	1400*	3000	1500–5000	14.2*	34.9	25–60%
**T Lymphocytes**	504*	**1620**	930–3450	36*	54*	62–69%
**TCD4+ Lymphocytes**	434*	1170	450–2000	31	39	30–40%
**TCD8+ Lymphocytes**	56*	360*	375–2000	4*	12*	25–32%
**NK cells**	560	390	120–750	40*	13	8–15%

* Out of range values

^a^ Patient age

## Discussion

The aim of this study was to evaluate NK cell phenotype and cytotoxic function in PMM2-CDG patients which might help to explain their tendency to recurrent/severe infections in early age. We studied twelve patients with different clinical severity and different *pmm2* mutations, three of them suffered from severe viral infections during their first year of life. Expression of surface activating, inhibitory receptors and adhesion molecules, known to be important during NK cell development and NK immune response, and the NK cytolytic activity were studied on blood lymphocytes and activated NK cells. We report here a significant increase of the frequency of responding NK cells that correlate with an increase of surface expression of CD54 (ICAM-1) adhesion molecule and NKG2D activating receptors together with an unbalanced expression of NKp46 and 2B4 receptors on activated NK cells from PMM2-CDG patients. Importantly, two severe patients exhibited an increased number of blood NK cells and a simultaneous decrease of TCD8^+^ lymphocytes after infection suggesting a direct association between the increased NK cell reactivity and the loss of TCD8^+^ cells in these patients.

The PMM2-CDG phenotype is very diverse, ranging from near-normal to very severe. This is primarily due to genetic heterogeneity however this does not explain why some patients with the same mutations can show different phenotypes and severity [23]. The genetic background of these patients is often invoked to explain this heterogeneity but this is a complex and poorly explored issue. In our work, we observed changes in the expression of CD54, NKG2D and NKp46 from a total of fourteen different glycoproteins tested. These changes did not correlate with patients’ clinical severity, genotype or the degree of glycosylation, illustrating the phenotypic heterogeneity of this pathology.

It is well established that N-glycosylation is a critical process that affects multiple mechanisms involved in immunity. N-glycosylation is essential for the interaction of NK activating receptors with their ligands and these interactions also affect the NK cytolytic activity. Thus, removal of N-linked carbohydrates by PNGase treatment or site-directed mutagenesis of predicted N-glycosylation sites severely compromised the specific binding of 2B4 activating receptor to its cellular ligand CD48. However, NK cell desialylation or inhibition of O-linked glycosylation resulted in increased 2B4-mediated lysis of CD48-expressing target cells [24]. Additionally, the degree of glycosylation impacts the ligand binding properties of human CD16 and NKp30 or the mouse Ly49D NK activating receptors [25–27]. N-glycosylation defects in human normal NK cells, studied using glycosylation inhibitors such as swainsonine, enhanced both NK cell cytotoxicity against tumor cells and the susceptibility of target cells to NK cytotoxicity [28]. GN4C, another modulator of cell glycosylation, altered the expression level of some glycosyltransferases, improved NK cell cytotoxic function against tumor cells and the expression of NKG2D mRNA [29]. According to these studies an increase in cytolytic activity and/or NKG2D surface expression is expected to occur in PMM2-CDG NK cells. Changes in glycosylation status may thus impair the recognition capability of NK cells and/or modify the recognition pattern of target cells in PMM2-CDG patients.

It is well stablished that the density of NKG2D, CD161 or NKp46 activating receptors on the NK cell surface correlates with the degree of NK cytotoxic activity against different targets, including autologous, allogeneic or xenogeneic target cells [30, 31]. In our study, increased frequencies of degranulating NK cells directly correlated with increased expression of surface CD54 and NKG2D on patient’s cells. The increased reactivity of PMM2-CDG NK cells could be related to the expression level of the surface receptors implicated in K562 target cell adhesion, detection and killing which, in the case of this target, are known to be at least NKG2D and NKp46 receptors [32]. However, the balanced expression of these activating receptors may also be of importance since it may influence the sensitivity of NK cells against each particular target, including healthy self- or virus infected cells. As far as we know, there are not previous studies related to the normal balanced expression of different activating receptors or the impact of this balance on NK cell cytotoxic activity against different targets. We found that, opposite to activated control cells, NKp46 or 2B4 expression negatively correlated with NKG2D expression on patient´s cells suggesting a possible imbalance expression of these killing receptors on activated cells that may have an effect on the balance between immune response and self-tolerance of PMM2-CDG NK cells.

Related to CD54 adhesion molecule, this is also an important participant in the immune response, constitutively present on the cell surface of a wide variety of cell types (fibroblasts, keratinocytes, endothelial and epithelial cells), that is upregulated in response to a number of inflammatory mediators, virus infection, oxidant stresses and pro-inflammatory cytokines [11]. It has been shown that glycosylation-deficient fibroblasts showed a reduced expression of CD54 [6, 16]. Rather than diminished expression of surface receptors we observed normal levels on peripheral blood leukocyte, except for the expression of CD54 on B lymphocytes. We think that these differences may be due to the cell type studied or the antibodies used to do the analysis, as shown for other cell markers such as CD14 [7]. Further analysis should be done to specify whether these differences are due to the use of antibodies which bind to a potentially hypoglycosylated epitope.

In normal leukocytes, CD54 is up-regulated during activation and its upregulation is indicative of level of cell activation [15]. Its function has been widely studied in NK target cells but little in NK effector cells. NK cell expressed CD54 strengthens the NK-target cell and the NK-leukocyte interactions and may influence several events during lytic interplay [33]. After activation, both control and patient cells up-regulated CD54 expression with mean values higher on patient NK cells. Altogether, our results may indicate a higher activation level and higher potential strength during NK-target or NK-leukocyte interactions of patient activated NK cells thus favouring target cell recognition and killing.

NK cell activity against target cells is controlled by the expression of NK activating and inhibiting receptors, However, NK cell reactivity is also modulated and shaped by processes such as development, education, priming, exposure to antigens or cytokines. The molecular mechanisms that govern responsiveness are not established [10]. Thus, MHC class I deficient mice generate hyporesponsive NK cells due to the lack of MHC class I-inhibitory receptors contact [34]. Additionally, in normal animals and humans, NK cells expressing receptors for self MHC exhibit greater responsiveness to stimulation than those NK cells which do not express them [35, 36]. The lack of NKp46 activating receptor expression in deficient mice is correlated with NK cell hyper-responsive [37]. Changes in NK cell reactivity can be also determined by inhibitory and activating receptor interactions during NK cell development. Since receptor-ligand interactions are also modulated by receptor and ligand glycosylation, it is reasonable to argue that the increased reactivity of PMM2-CDG NK cells could be stablished during NK cell development. In this sense, we found a higher frequency of fresh responsive NK cells from P2 and P5 severe patients compared to healthy controls. Hyper-reactivity displayed by PMM2 NK cells cannot be exclusively defined by the levels of CD226 expression as shown by normal cells [22] and the expression and function of other receptors such as inhibitor receptors could be involved. Further studies are needed to address whether impaired N-glycosylation might affect modulation of cytolytic function on NK cells.

A phenotype of severe viral infections may be related to T and/or NK cell dysfunction. Reduction of CD8^+^ T cells (Fig 6) was associated with a notable increase of NK cell counts in our severe patients suggesting that cytotoxic adaptive response may be compromised during their first year of life. An increase of mature NK cell cytotoxic activity would suggest a higher protection from infections and a compensation for a possible TCD8 dysfunction. However, according to different studies, this fact is controversial since NK cell cytotoxic activity can have positive or negative effects on pathogen protection. Thus, NK cell or NK cell function deficiency is associated with susceptibility to viral infections [38]. More interesting, hyperactivity of mouse NK cells may provoke the reduction of memory T cell numbers during a viral or bacterial infection [37], i. e., a high NK lytic activity might be associated with a deficient immune response against microorganisms. Moreover, the reduction of NK cells augmented memory CD8^+^ T cells in a model of human infection [39]. Particularly, we found a general increase in the cytotoxic activity in PMM2-CDG NK cells and an imbalance expression of NKG2D, NKp46 and 2B4 activating receptors. Additionally, we found elevated numbers of NK cells and low numbers of T cells in the blood of the patients P2 and P3, both affected by several moderate to severe viral infections. In this regard, there is evidence from mouse and human studies that NK cells exhibit immunoregulatory functions by interacting with controlling populations of different immune cells such as antigen presenting cells, epithelial stromal cells or activated TCD4^+^ and TCD8^+^ cells (extensively reviewed by Crome et al. [9]). Several studies have demonstrated the direct killing of activated T cells mediated by NKG2D [40, 41] or NKp46 activating receptors [23] on NK cells. Poggy et al. [42] showed the killing of autologous antigen presenting and stromal cells only by activated human NK cells that was dependent on LFA1/CD54 interaction and the activation of NKp30, NKp46 and NKG2D killing receptors. In *in vivo* studies, NK cell depletion promoted LCMV-induced CD8^+^T cell immunity with the involvement of both perforin and NKG2D [43]. Altogether, these studies show that NK cells can be crucial in controlling infections not only by a direct elimination of infected cells, but also by altering the number and function of specific T cells. Thus, the increase in NK cytotoxic activity could contribute to the higher frequency of infections in some PMM2-CDG patients. Moreover, uncontrolled NK cytotoxic activity could reduce the adaptive immune response during infection explaining at least partially the high susceptibility of PMM2-CDG patients to suffer from severe infections in the first years of life. Further studies of NK cells and TCD8+ lymphocytes are needed in a larger number of patients with PMM2-CDG and in other CDG.

## Supporting Information

S1 Fig (A) Isotherms obtained by non-linear regression analysis from frequencies of degranulation (α) of activated NK cells co-cultured with K562 target cells at different effector/target ratios (R), plotted as α versus R, that defines reactivity of activated NK cells and were used to calculate the frequency of degranulated cells at E/T ratio 1:1. (B) Isotherms obtained by linear regression analysis by plotting 1/α vs 1/R (i.e., vs. 1/R (T:E ratio)) used to calculate the maximal frequency of activated NK cells against K562 targets on patient’s and control samples, being 1/α_max_ the intersection point of this plot. Equation and correlation coefficient are shown for each isotherm.
(TIF)

S2 Fig Expression of externalized CD107a glycoprotein on activated NK from PMM2-CDG patients and control subjects. The study was done by flow cytometry on degranulated NK cells used in the degranulation assay at the highest E/T ratio. Values are expressed as % MFI *vs* that observed in control subjects.
(TIF)

S1 Table Cell types and molecules evaluated in this study. The monoclonal antibodies used in flow cytometry are also listed.
(PDF)

S2 Table Distribution of plasma sialotransferrin fractions from PMM2-CDG patients. (PDF)

S3 Table Killing activity (E/T 0.5:1), degranulation and perforin levels in P3, P5 and P9 PMM2-CDG patients. (PDF)

S4 Table **(A) Expression of CD226 regulatory molecule in several PMM2-CDG patients evaluated by flow cytometry. (B) Percentages of blood lymphocytes expressing CD226 regulatory molecule levels from several PMM2-CDG patients**.
(PDF)

S5 Table Expression of CD11a and CD50 adhesion molecules in several PMM2-CDG patients evaluated by flow cytometry. (PDF)
