# Correction: Natural Killer Cell Receptors and Cytotoxic Activity in Phosphomannomutase 2 Deficiency (PMM2-CDG)

**DOI:** 10.1371/journal.pone.0165901

**Published:** 2016-10-27

**Authors:** Roberto García-López, María Eugenia de la Morena-Barrio, Laia Alsina, Belén Pérez-Dueñas, Jaak Jaeken, Mercedes Serrano, Mercedes Casado, Trinidad Hernández-Caselles

The following information is missing from the Funding section: The work was supported by national grants PI14/00021 from the National Plan on I+D+I, cofinanced by ISC-III (Subdirección General de Evaluación y Fomento de la Investigación Sanitaria) and FEDER (Fondo Europeo de Desarrollo Regional).
