# Supplementary figures and images for: Natural Killer Cell Receptors and Cytotoxic Activity in Phosphomannomutase 2 Deficiency (PMM2-CDG)
Source: PLoS One. 2016 Jul 14;11(7):e0158863. doi: 10.1371/journal.pone.0158863 (PMC4944953; doi:10.1371/journal.pone.0158863)

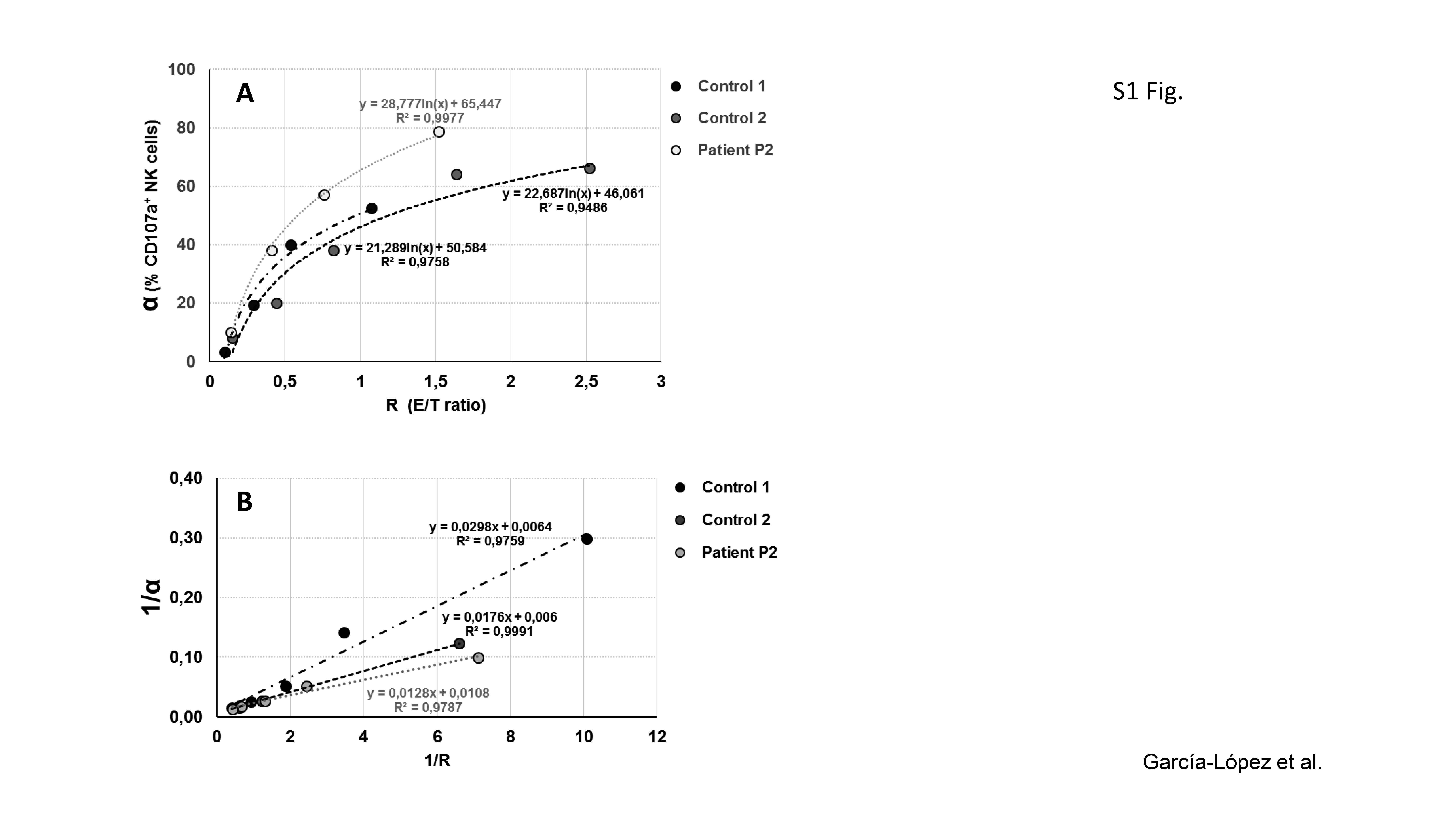

Supplement: S1 Fig — (A) Isotherms obtained by non-linear regression analysis from frequencies of degranulation (α) of activated NK cells co-cultured with K562 target cells at different effector/target ratios (R), plotted as α versus R, that defines reactivity of activated NK cells and were used to calculate the frequency of degranulated cells at E/T ratio 1:1. (B) Isotherms obtained by linear regression analysis by plotting 1/α vs 1/R (i.e., vs. 1/R (T:E ratio)) used to calculate the maximal frequency of activated NK cells against K562 targets on patient’s and control samples, being 1/αmax the intersection point of this plot. Equation and correlation coefficient are shown for each isotherm. (TIF) [file pone.0158863.s001.tif]

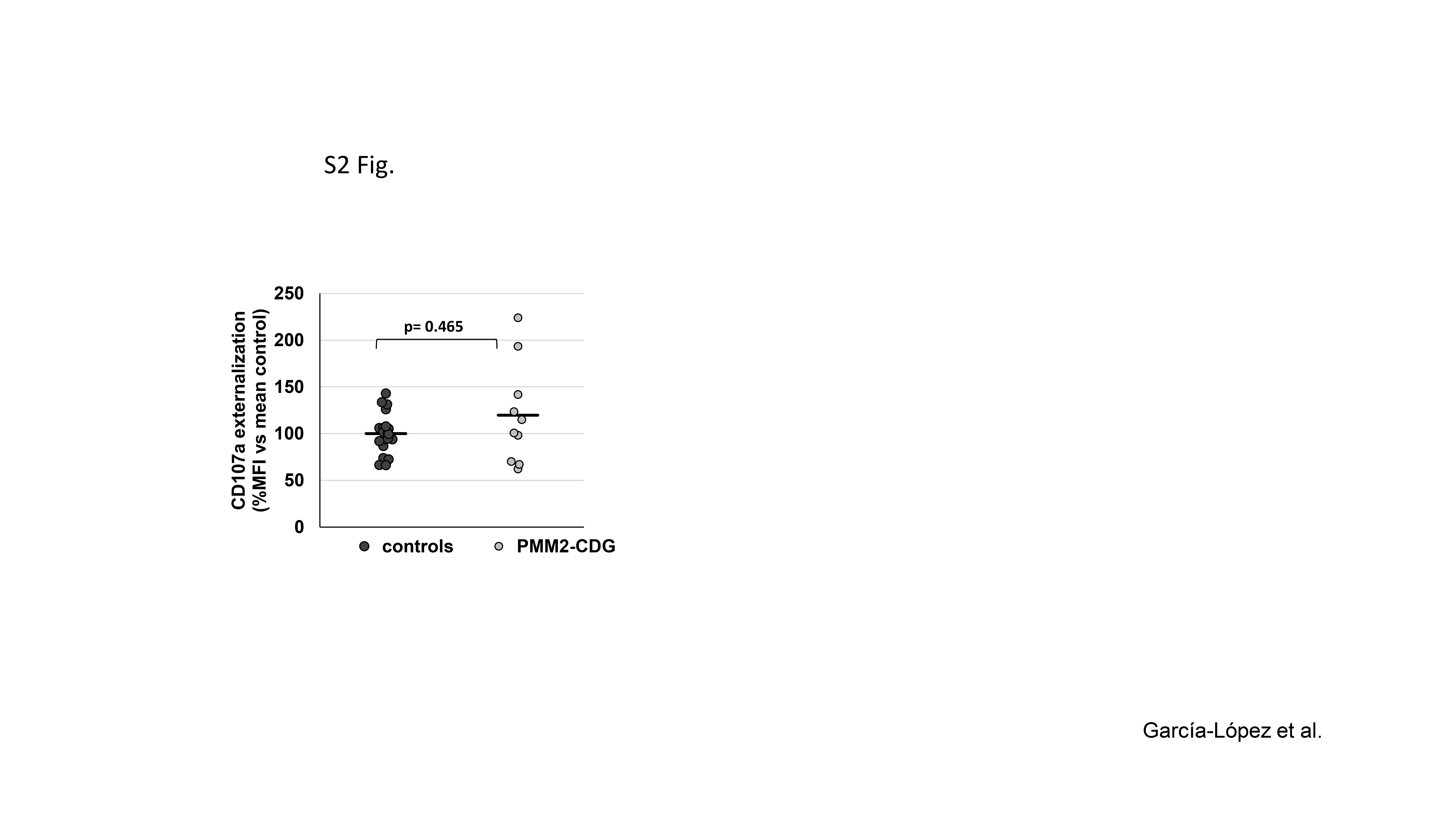

Supplement: S2 Fig — The study was done by flow cytometry on degranulated NK cells used in the degranulation assay at the highest E/T ratio. Values are expressed as % MFI vs that observed in control subjects. (TIF) [file pone.0158863.s002.tif]
