# Supplementary material for: Natural Killer Cell Receptors and Cytotoxic Activity in Phosphomannomutase 2 Deficiency (PMM2-CDG)
Source: PLoS One. 2016 Jul 14;11(7):e0158863. doi: 10.1371/journal.pone.0158863 (PMC4944953; doi:10.1371/journal.pone.0158863)
Supplement: S1 Table — The monoclonal antibodies used in flow cytometry are also listed. (PDF) [file pone.0158863.s003.pdf]

S1 Table. Cell types and molecules evaluated in this study. The monoclonal antibodies used in flow cytometry are also listed.

Cell type	Molecule	MoAb	Source
T lymphocytes	CD3	SK7 (FITC)	BD Pharmingen
	CD54	HA58 (PE)	BD Pharmingen
	CD56	B159 (PE)	BD Pharmingen
NK cells	CD16	3G8 (PE)	BD Pharmingen
	CD54	HA58 (PE)	BD Pharmingen
	CD56	B159 (PE)	BD Pharmingen
	Siglec-7	6-434 (PE)	BioLegend
T and NK lymphocytes	CD226	11A8	BioLegend
	NKG2D	1D11	eBioscience
	NKp46	195314	R&D Systems
	2B4	C1.7	Beckman-Coulter
	NKG2A	Z199	Dr. A. Moretta (Geneva, Italy)
	CD11a	TP1/11	Dr. F. Sánchez-Madrid (Madrid, Spain)
	ICAM-3	HP2/19	Dr. F. Sánchez-Madrid (Madrid, Spain)
Monocytes	CD14	61D3	eBioscience
