# Supplementary material for: Natural Killer Cell Receptors and Cytotoxic Activity in Phosphomannomutase 2 Deficiency (PMM2-CDG)
Source: PLoS One. 2016 Jul 14;11(7):e0158863. doi: 10.1371/journal.pone.0158863 (PMC4944953; doi:10.1371/journal.pone.0158863)
Supplement: S2 Table — (PDF) [file pone.0158863.s004.pdf]

S2 Table. Distribution of plasma sialotransferrin fractions from PMM2-CDG patients.

	Transferrin (% of total)*			Clinical severity
**Patients**	**Asialo-**	**Disialo-**	**Trisialo-**	
**P1**	21.47	38.17	1.74	Severe
**P2**	17.6	41.61	1.33	Severe
**P3**	11.05	45.82	2.99	Severe
**P4**	8.48	32.4	0.78	Severe
**P5**	23.58	44.06	0,00	Severe
**P7**	27.6	40.5	1.7	Mild/Moderate
**P8**	9.4	36.1	1.7	Mild/Moderate
**P9**	6	30.1	3.2	Mild/ Moderate
**P10**	4.24	19.32	2.27	Mild
**P11**	12.7	35.7	4.2	Mild
**P12**	8.7	32.6	4.8	Mild
**Control pool**	1.8	1.2	8.7	-

*Area of asialo-, disialo- and trisialo-transferrin HPLC peaks.
