# Supplementary material for: Natural Killer Cell Receptors and Cytotoxic Activity in Phosphomannomutase 2 Deficiency (PMM2-CDG)
Source: PLoS One. 2016 Jul 14;11(7):e0158863. doi: 10.1371/journal.pone.0158863 (PMC4944953; doi:10.1371/journal.pone.0158863)
Supplement: S3 Table — (PDF) [file pone.0158863.s005.pdf]

S3 Table. Killing activity (E/T 0.5:1), degranulation and perforin levels in P3, P5 and P9 PMM2-CDG patients.

Patients	**Target cell killing** (% vs control mean)	**CD107a** (% MFI vs control mean)*	**Perforin** (relative levels)[a]
P3	124.9	100.7	1.3
P5	108.3	224.0	1.5
P9	76.3	98.2	n.d.
Controls	100.0 ± 31.4**	100.0 ± 21.0	1.1±0.3

* CD107a externalized during NK cell cytotoxicity assays. ** n=7

n.d.: not determined

[a] perforin/erk-2 ratio
