# Supplementary material for: Natural Killer Cell Receptors and Cytotoxic Activity in Phosphomannomutase 2 Deficiency (PMM2-CDG)
Source: PLoS One. 2016 Jul 14;11(7):e0158863. doi: 10.1371/journal.pone.0158863 (PMC4944953; doi:10.1371/journal.pone.0158863)
Supplement: S4 Table — (A) Expression of CD226 regulatory molecule in several PMM2-CDG patients evaluated by flow cytometry. (B) Percentages of blood lymphocytes expressing CD226 regulatory molecule levels from several PMM2-CDG patients. (PDF) [file pone.0158863.s006.pdf]

S4A Table. Expression of CD226 regulatory molecule in several PMM2-CDG patients evaluated by flow cytometry.

	CD226 expression on lymphocytes (% MFI vs control mean)					
	Blood lymphocytes				Activated lymphocytes	
**Patients**	**$CD3^{+}$ $CD226^{low}$**	**$CD3^{+}CD226^{high}$**	**$CD3^{-}CD19^{-}$ $CD226^{low}$**	**$CD3^{-}CD19^{-}$ $CD226^{high}$**	**$CD3^{+}$**	**$CD3^{-}$**
**P2***	n.d.	n.d.	n.d.	n.d.	103.3	98.4
**P3***	126.1	75.1	120.8	114.5	104.4	138.5
**P5***	108.4	94.2	126.7	114.9	112.4	83.2
**P6**	105.2	103.7	98.9	87.2	84.0	72.7
**P9**	n.d.	n.d.	n.d.	n.d.	100.7	92.8
patient´s mean ± SD	113.3 ± 11.3	91.3 ± 14.6	115.5 ± 14.6	105.5 ± 15.8	101.0 ± 10.4	97.1 ± 25.1
Controls mean ± SD**	100.0 ± 3.9	100.0 ± 4.9	100.0 ± 6.6	100.0 ± 5.2	100.0 ± 15.9	100.0 ± 11.7

* Severe patients
n.d. : not determined
** n=9

S4B Table. Percentages of blood lymphocytes expressing CD226 regulatory molecule levels from several PMM2-CDG patients.

	CD226+ blood lymphocytes(%)			
**Patients**	**$CD3^{+}$ $CD226^{low}$**	**$CD3^{+}CD226^{high}$**	**$CD3^{-}CD19^{-}$ $CD226^{low}$**	**$CD3^{-}CD19^{-}$ $CD226^{high}$**
**P3***	31.9	60.7	42.5	57.5
**P5***	21.0	64.3	**84.9**	**15.1**
**P6**	33.2	57.9	53.3	46,7
patient´s mean ± SD	28.7 ± 6.7	61.0 ± 3.2	60.2 ± 22.0	39.8 ± 22.0
Controls mean ± SD**	29.0 ± 9.0	58.6 ± 10.2	46.2 ± 17.0	53.8 ± 17.0

* Severe patients
n.d. : not determined
* n=7
