# Supplementary material for: Natural Killer Cell Receptors and Cytotoxic Activity in Phosphomannomutase 2 Deficiency (PMM2-CDG)
Source: PLoS One. 2016 Jul 14;11(7):e0158863. doi: 10.1371/journal.pone.0158863 (PMC4944953; doi:10.1371/journal.pone.0158863)
Supplement: S5 Table — (PDF) [file pone.0158863.s007.pdf]

**<u>S5 Table</u>. Expression of CD11a and CD50 adhesion molecules in several PMM2-CDG patients evaluated by flow cytometry.**

| | **Adhesion molecules expression (% MFI vs control mean)** | |
|---|---|---|
| **Patients** | **CD11a** | **CD50 (ICAM-3)** |
| P2* | 121.6 | n.d. |
| P3* | 76.7 | 77.7 |
| P5* | 80.4 | 79.4 |
| P6 | 107.5 | 111.2 |
| P9 | 129.5 | 88.2 |
| Patient´s mean ± SD | 103.1 ± 23.8 | 89.1 ± 15.4 |
| Control mean ± SD | 100.0 ± 21.2 | 100.0 ± 9.2 |

* severe patients

n.d. : not determined
